# Childhood vaccinations and adult schooling attainment: Long-term evidence from India's Universal Immunization Programme

**DOI:** 10.1016/j.socscimed.2020.112885

**Published:** 2020-04

**Authors:** Arindam Nandi, Santosh Kumar, Anita Shet, David E. Bloom, Ramanan Laxminarayan

**Affiliations:** a Center for Disease Dynamics, Economics & Policy, 1400 Eye St. NW, Suite 500, Washington, DC, 20005, USA; b Department of Economics and International Business, College of Business Administration, Sam Houston State University, SHB 237F, Huntsville, TX, 77340, USA; c Johns Hopkins Bloomberg School of Public Health, 415 N. Washington Street, Baltimore, MD, 21231, USA; d Department of Global Health and Population, Harvard T.H. Chan School of Public Health, 665 Huntington Avenue, Building I 12th Floor, Suite 1202, Boston, MA, 02115, USA; e Center for Disease Dynamics, Economics & Policy, B-25, Lajpat Nagar II, New Delhi, Delhi 110024, India; f Princeton Environmental Institute, Princeton University, Princeton, NJ, 08544, USA

**Keywords:** India, Universal Immunization Programme, UIP, Child development, Vaccine, Schooling attainment

## Abstract

Routine childhood vaccines are among the most cost-effective life-saving interventions. In addition, vaccines have been linked with reduced stunting and improved health and other outcomes in later life. However, evidence on such long-term benefits remain inadequate. In this study, we examined the associations between the initiation and implementation of the Universal Immunization Programme (UIP) in India and schooling attainment among adults. We obtained district-level data on the rollout of the UIP in 1985–1990 and matched those with data from the National Family Health Survey of India, 2015–2016. Adults who were born in the five years before and after the rollout period (1980–1995) and always lived in the same location were included in the analysis (n=109,908). We employed household, village or city ward, district, and state fixed-effects linear regression models, which incorporated a wide range of socioeconomic and demographic indicators and community-level infrastructure, amenities, and access to healthcare. We compared schooling attainment in years among individuals who were born during or after the UIP was implemented in their districts (intervention group) with those who were born before UIP implementation (control group). In household fixed-effects analysis, intervention group adults attained 0.18 (95% confidence interval [CI]: 0.02, 0.33; p<0.05) more schooling grades as compared with control group adults from the same household. In village or city ward, district, and state fixed-effects analysis, intervention group adults attained 0.23 (95% CI: 0.13, 0.32; p<0.001), 0.29 (95% CI: 0.19, 0.38; p<0.001), and 0.25 (95% CI: 0.1, 0.39; p<0.01) additional schooling grades, respectively, compared to the control group. In subgroup analyses, positive associations between UIP implementation and schooling grades were observed among women and among rural, urban, and richer households. Our results support the association of vaccines with improved school attainment.

## Highlights

• Vaccines could provide a broad range of health and non-health benefits.
• We examined the link between childhood vaccination and adult schooling in India.
• India's UIP was associated with 0.2–0.3 additional schooling grades.

## 1 Introduction

In 1990–2015, the annual number of deaths among children under the age of five years fell from 12.7 million to 5.9 million globally and from 3.4 million to 1.2 million in India (You et al., 2015). Routine childhood vaccinations have contributed substantially to improved child survival rates, preventing an estimated 2–3 million deaths each year globally (World Health Organization, 2018). They are among the most cost-effective life-saving interventions, especially in the context of low- and middle-income countries (LMICs) (Ozawa et al., 2012).

In addition to morbidity, mortality, and the economic cost of treatment, vaccine-preventable diseases are known to cause stunting in childhood, which can lead to poor growth, poor adult health, and diminished learning capacity and economic productivity (Almond and Currie, 2011; Currie and Vogl, 2013a; Dewey and Begum, 2011). Routine childhood vaccinations can not only lessen the immediate burden of diseases, but could also reduce stunting and thereby improve health and other outcomes over the life cycle (Anekwe et al., 2015; Anekwe and Kumar, 2012; Bloom et al., 2012; Canning et al., 2011; Driessen et al., 2015; Nandi et al., 2019c; Nandi and Shet, 2020; Upadhyay and Srivastava, 2017).

However, evidence linking childhood vaccination to later life outcomes in LMICs, which is based on small-scale studies from Ethiopia, Bangladesh, India, the Philippines, South Africa, and Vietnam, remains inadequate. Longitudinal data from the Young Lives survey on approximately 2000 children each in Ethiopia, India, and Vietnam, have been used to link measles vaccination at ages 6–18 months of life with 0.1–0.2 higher anthropometric z-scores, 1.7–4.5 percentage points higher scores on standardized cognitive tests, and 0.2–0.3 additional schooling grades at ages 7–8 and 11–12 years (Nandi et al., 2019c). Data from the same source have been used in two other studies that found an association between *Haemophilus influenzae* type B (Hib) vaccination before the age of six years and improved later life outcomes in India. The first study found that Hib-vaccinated children were 24% less likely to be stunted and 21% less likely to be underweight at ages 4–6 years (Upadhyay and Srivastava, 2017). The second study estimated that Hib-vaccinated children had 0.2–0.3 higher height-for-age z-scores, 3.2–4.8 percentage point higher cognitive test scores, and 0.1–0.2 more schooling grades at ages 11–12 and 14–15 years (Nandi et al., 2019b).

A South African study based on data on 4783 children of age 6–11 years found that those who received the measles vaccine attained 0.2 more schooling grades on average as compared with their siblings (Anekwe et al., 2015). A longitudinal study of 1975 children of age 10–11 years in the Philippines found that those who were fully immunized within the first two years of life had 0.5 standard deviation higher cognitive test scores (Bloom et al., 2012).

Among large-scale studies, a phased introduction of the measles vaccine among 35,000 children in the Matlab district of Bangladesh was linked with 7.4 percentage points higher school enrolment among 8–16 year old boys but not girls (Driessen et al., 2015). A nationally representative study of 49,000 children under the age of 4 years associated exposure to India's routine childhood vaccination program (Universal Immunization Programme, or UIP) during the first year of life with 0.3–0.5 higher height-for-age and weight-for-age z-scores (Anekwe and Kumar, 2012).

The current literature on the long-term benefits of childhood vaccines has some important limitations. While the efficacy of a vaccine in reducing the immediate burden of disease is context independent, its broader benefits over the life cycle may be mediated through underlying socioeconomic conditions, rates of undernutrition, and access to and quality of healthcare and schooling—which vary across settings. Therefore, the findings of previous studies may not be generalizable to other LMICs. Regional studies, such as those for the measles and Hib vaccines in the state of Andhra Pradesh in India and measles vaccine in the Matlab district of Bangladesh and KwaZulu-Natal province of South Africa may not be externally valid even within the respective country (Anekwe et al., 2015; Driessen et al., 2015; Nandi et al., 2019c). Finally, to the best of our knowledge, no study has yet examined the long-term benefits of childhood vaccinations among individuals over the age of 16 years.

Whether childhood vaccinations can improve schooling outcomes in adulthood, and nationally, remains unknown for India. In this study, we investigated the long-term associations between vaccination through the UIP and schooling attainment of Indian adults, using national household survey data and information on the district-wise rollout of the UIP in 1985–1990 in India. Employing household and community fixed-effects regression models, we compared the completed schooling grades of 20–36 year old adults who were born before and after implementation of the UIP.

## 2 Data and methods

### 2.1 Data: the UIP and its phased introduction in 1985–1990

India launched the World Health Organization's (WHO) Expanded Programme of Immunization (EPI) in 1978 with the introduction of Bacillus Calmette–Guérin (BCG), oral polio vaccine (OPV), diphtheria–tetanus–pertussis (DPT), and typhoid–paratyphoid vaccines for children (Lahariya, 2014; Vashishtha and Kumar, 2013). The typhoid–paratyphoid vaccine was removed from the Indian EPI in 1982, and the maternal tetanus toxoid (TT) vaccine was added in 1983 (Lahariya, 2014). In 1985, the program was redesigned and renamed the Universal Immunization Programme. The goal of the UIP was to improve service delivery, establish a cold chain, implement monitoring and evaluation mechanisms at the district level, and eventually achieve national self-sufficiency in manufacturing vaccines and cold chain equipment (Lahariya, 2014). The UIP included four childhood vaccines for infants: one dose each of measles and BCG, three doses of OPV, and three doses of DPT. The goal of the program was to cover all pregnant women (with TT) and 85% of infants by 1990 (Lahariya, 2014).

The UIP was first implemented in 30 districts in 1985–1986 and scaled up across all 353 districts of India by 1990 in a phased-annual manner (Anekwe and Kumar, 2012; Lahariya, 2014). Each phase's districts were selected based on characteristics that were either time invariant or had a slow rate of change (e.g., health infrastructure) (Anekwe and Kumar, 2012; Kumar, 2009). The program was prioritized in districts with higher availability and quality of public health infrastructure because they were likely more capable of achieving and maintaining target vaccination rates (Anekwe and Kumar, 2012; Lahariya, 2014).

One of the coauthors of this study collected the district-level rollout data for the UIP (1985–1990) from the Ministry of Health and Family Welfare of India. These data have been used in a previous study (Anekwe and Kumar, 2012).

### 2.2 Data: National Family Health Survey of India 2015–2016

We combined the UIP rollout data by district with data from the National Family Health Survey of India 2015–2016 (NFHS-4) (International Institute for Population Sciences and ICF, 2017). NFHS-4 was a cross-sectional survey of 601,509 households and 2.87 million individuals from all states and union territories of India. The main focus of the survey was health and family welfare, and it collected data on various indicators such as socioeconomic and demographic characteristics; anthropometry; and biomarkers related to anemia, hypertension, and diabetes (International Institute for Population Sciences and ICF, 2017). A separate questionnaire for all 15–49 year old women collected detailed data on reproductive health, fertility and family planning, child care, nutrition, vaccination, domestic violence, and knowledge of HIV/AIDS. Another questionnaire for 15–54 year old men—administered in a nationally representative subsample of 15% of households—collected data on topics such as marriage, employment, family planning and fertility preferences, and gender role perceptions.

India had 353 districts when the UIP was implemented in 1985–1990. Over time, with the creation of new states, bifurcation of districts, and adjustment of administrative borders, the number of districts increased to 722 in 2019 (Government of India, 2019). We retrospectively matched the districts following a two-step process. The UIP-era districts were matched with 393 districts in the National Family Health Survey of India 1992–1993 (NFHS-1) in a previous study (Anekwe and Kumar, 2012). We used information from published administrative documents (e.g., state and district web portals) to match the NFHS-1 districts with the Indian Census of 2001, which had 593 districts, and then with NFHS-4, which had 640 districts (based on the Census of 2011) (Office of the Registrar General and Census Commissioner, 2011; Office of the Registrar General and Census Commissioner, India, n.d.).

**Fig. 1:**
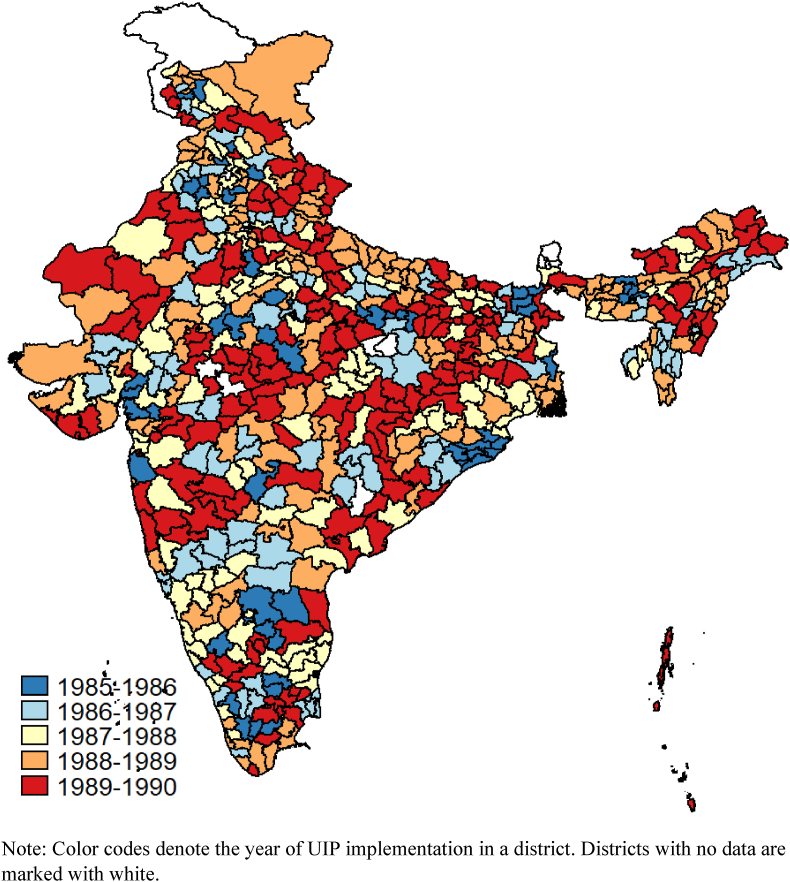
District-wise rollout of India's UIP in 1985–1990. Note: Color codes denote the year of UIP implementation in a district. Districts with no data are marked with white. (For interpretation of the references to colour in this figure legend, the reader is referred to the Web version of this article.)

We were able to match 621 NFHS-4 districts with the 353 UIP-era districts. The remaining 19 districts from Chhattisgarh (1), Madhya Pradesh (4), Delhi (9), Punjab (1), and Sikkim (4) could not be matched. Fig. 1 shows the district-wise rollout of the UIP across India, using data from NFHS-4 districts.

The questionnaires for women and men in NFHS-4 collected information on the length of residence of each individual. Among the 699,686 women of age 15–49 years and 112,122 men of age 15–54 years, 23% and 70% respectively reported living in the same location (village or neighborhood) since birth. We included only these nonmigrant individuals in our analysis, as the survey did not report the previous place of residence for migrants. We compared the birth year of each individual with the year of UIP implementation in the district and included those born during or after the UIP implementation year in the intervention group. The control group included those who were born before the UIP was implemented in the district.

The outcome variable of our analysis was the grades of schooling completed by individuals, which ranged from 0 to 20.

### 2.3 Empirical analysis: household fixed-effects regression model

As discussed earlier, the rollout sequence of the UIP was positively associated with the capacity of the public healthcare system at the district level (Anekwe and Kumar, 2012; Kumar, 2009). Therefore, systematic differences in the characteristics of the intervention and control groups could bias ordinary least squares estimates of the relationship between exposure to the UIP and future schooling attainment. To mitigate such biases, we used a household fixed-effects regression model that compared intervention and control individuals within each household. We linearly regressed grades of schooling attainment on individuals’ intervention status (i.e., whether born during, or after, the year UIP was implemented in their district of residence). The model covariates included individual characteristics: age in years, squared age, sex (whether female), and marital status (whether single). Intrahousehold resource allocation, which could affect human capital development and long-term schooling outcomes, was captured by including indicators of relationship to the household head (self, spouse, child, in-law, or grandchild) among the covariates.

To reduce systematic differences among members within a given household, we included only individuals who were born during the UIP implementation era (1985–1990) and in the five years before (1980–1984) and after (1991–1995) that period, i.e., those who were 20–36 years old at the time of the NFHS-4 survey.

The household fixed effects included in the model mitigated potential confounding factors shared by members of a household, such as location, standard of living, religion, caste, and access to and quality of public healthcare including child immunization. Cohort-specific factors that differed across members of the same household, e.g., changes in local healthcare or schooling infrastructure over time, were captured by the age and squared age variables. The standard errors of the regression model were robust clustered at the household level.

### 2.4 Sensitivity analysis: Village, district, and state fixed-effects regression models and expanded age range

We tested the sensitivity of our results with three additional regression models. The first was a village fixed-effects model that compared schooling outcomes of individuals within the same village (or city ward, for urban areas). The covariates of this regression included individual characteristics as discussed earlier, along with indicators of location (rural vs. urban), socioeconomically disadvantaged caste groups (scheduled caste, scheduled tribe, or other backward classes), religion (Muslim, Christian, or Sikh), age of the household head in years, and whether the household head was female.

This model's household standard of living was captured through a wealth index, which we created as a composite index of ownership of assets such as TV, radio, bicycle, and car, along with indicators of living conditions such as construction quality; number of rooms; and availability of toilet, drinking water, and electricity (Filmer and Pritchett, 2001; Pollitt et al., 1993). We divided the wealth index into quintiles and included indicators of the top four quintiles among the model covariates. We also included village fixed effects, which controlled for factors that all households in the village shared, such as infrastructure, amenities, and access to and quality of public healthcare. Standard errors were robust clustered at the village level.

We repeated our analysis with two additional models that included district fixed effects and state fixed effects, respectively, instead of village fixed effects. The covariates of these models included the same individual and household characteristics as described previously. Standard errors of these models were robust clustered at district and state levels, respectively.

Finally, we conducted additional analysis for an expanded age range: adult women of age 18–49 years and adult men of age 18–54 years who lived in the same location since birth. We used all previously described four regression model specifications—household, village, district, and state fixed-effects—and reported results separately.

For all models, in addition to estimating the associations between schooling attainment and intervention status in the national sample, we conducted analyses separately for men and women. Because most marriages in India are patrilocal (Chadda and Deb, 2013), i.e., married women live with their in-laws, our subsample of women included both unmarried women who lived in a household since birth and married women who grew up elsewhere (in the same village or neighborhood) but moved to the household after marriage. Therefore, we conducted analysis also for the subsample of unmarried women.

Additional subsample analyses included those for households in rural and urban areas, general caste (i.e., not socioeconomically disadvantaged), scheduled caste (SC) or scheduled tribe (ST), other backward classes (OBC), and households in the first (poorest) and the fifth (richest) wealth quintiles. We used STATA version 14.2 for analysis and considered P < 0.05 for statistical significance.

## 3 Results

### 3.1 Characteristics of the study sample

Table 1 presents the summary statistics of our study sample. There were 110,067 men and women of age 20–36 years who had lived in the same place since birth. Of them, 61% were in the intervention group (born when or after the UIP was implemented in the district), and the rest were in the control group (born before the UIP). The average schooling attainment in the two groups was 10.3 and 8.1 grades respectively, with the difference being statistically significant.

**Table 1:** Summary statistics of Indians born in 1980–1995 (20–36 year old).

	Intervention		Control		Difference in means
	Mean	SD	Mean	SD	
Grades of schooling attainment	10.29	4.60	8.12	5.18	2.17**
Whether female	0.71	0.45	0.49	0.50	0.22**
Age in years	22.68	2.31	30.85	3.54	−8.17**
Whether single (unmarried)	0.76	0.42	0.26	0.44	0.50**
Relationship head of household—self	0.04	0.20	0.26	0.44	−0.22**
Relationship head of household—spouse	0.04	0.19	0.18	0.38	−0.14**
Relationship head of household—child	0.79	0.41	0.45	0.50	0.34**
Relationship head of household—in-law	0.03	0.16	0.05	0.21	−0.02**
Relationship head of household—grandchild	0.04	0.21	0.01	0.10	0.03**
Age of household head in years	51.64	12.23	48.33	15.11	3.31**
Whether household head is female	0.14	0.35	0.14	0.35	0.001**
Rural household	0.69	0.46	0.71	0.45	−0.02**
SC household	0.17	0.38	0.17	0.37	0.006*
ST household	0.20	0.40	0.26	0.44	−0.06**
OBC household	0.35	0.48	0.33	0.47	0.02
Muslim household	0.18	0.38	0.14	0.34	0.04**
Christian household	0.10	0.30	0.13	0.33	−0.03**
Sikh household	0.03	0.18	0.02	0.13	0.01**
Household belongs to second wealth quintile	0.18	0.39	0.19	0.39	−0.01**
Household belongs to third wealth quintile	0.22	0.41	0.22	0.41	−0.0003**
Household belongs to fourth wealth quintile	0.22	0.42	0.22	0.41	0.004**
Household belongs to fifth (richest) wealth quintile	0.25	0.43	0.22	0.42	0.03**
Sample size	67,097		42,970		

Note: Data are from the National Family Health Survey of India 2015–2016. The sample consists of 20–36 year old individuals, all of whom had lived in the same location since birth. Difference is the difference in means for the intervention group versus the control group. SD denotes standard deviation. *P < 0.05, **P < 0.01.

Except for the proportion of other backward classes, all other variables were significantly different on average between the intervention and control groups. Compared with the control group, the intervention group had a larger proportion of women and unmarried individuals, was younger, and was more likely to be children or grandchildren of the household head. The intervention group also had older household heads and was richer, more from urban areas, and less likely to belong to tribal groups, as compared with the control group.

Table 2 presents comparisons between nonmigrants and migrants (who were excluded) in our data. Except for the proportion of rural households, all socioeconomic and demographic characteristics were statistically different between the two groups. Nonmigrants had a larger proportion of women and unmarried individuals and were from richer households as compared with migrants. They also attained more schooling grades.

**Table 2:** Summary statistics of Indians born in 1980–1995 (20–36 year old), migrants and nonmigrants.

	Nonmigrants		Migrants		Difference in means
	Mean	SD	Mean	SD	
Grades of schooling attainment	9.45	4.95	8.01	5.09	1.44**
Whether female	0.63	0.48	0.49	0.50	0.14**
Age in years	25.87	4.90	27.76	4.84	−1.89**
Whether single (unmarried)	0.57	0.50	0.23	0.42	0.34**
Relationship head of household—self	0.13	0.33	0.18	0.39	−0.06**
Relationship head of household—spouse	0.09	0.29	0.24	0.43	−0.15**
Relationship head of household—child	0.66	0.47	0.34	0.47	0.32**
Relationship head of household—in-law	0.03	0.18	0.17	0.37	−0.13**
Relationship head of household—grandchild	0.03	0.17	0.01	0.11	0.02**
Age of household head in years	50.35	13.52	46.32	14.70	4.03**
Whether household head is female	0.14	0.35	0.12	0.32	0.02**
Rural household	0.70	0.46	0.70	0.46	0.00
SC household	0.17	0.38	0.18	0.39	−0.01**
ST household	0.23	0.42	0.18	0.38	0.05**
OBC household	0.34	0.47	0.39	0.49	−0.05**
Muslim household	0.16	0.37	0.14	0.34	0.03**
Christian household	0.11	0.31	0.07	0.25	0.04**
Sikh household	0.03	0.16	0.02	0.15	0.004**
Household belongs to second wealth quintile	0.18	0.39	0.19	0.39	−0.01**
Household belongs to third wealth quintile	0.22	0.41	0.21	0.41	0.01**
Household belongs to fourth wealth quintile	0.22	0.42	0.21	0.41	0.01**
Household belongs to fifth (richest) wealth quintile	0.24	0.43	0.22	0.41	0.02**
Sample size	110,067		680,986		

Note: Data are from the National Family Health Survey of India 2015–2016. The sample consists of 20–36 year old individuals. Nonmigrants lived in the same place since birth, while migrants did not. Difference is the difference in means for the nonmigrant group versus the migrant group. SD denotes standard deviation. *P < 0.05, **P < 0.01.

### 3.2 Household fixed-effects regression results

Table 3 presents results from the fixed-effects regression models for 20–36 year old adults. In household fixed-effects analysis, schooling attainment among intervention group individuals was 0.18 (95% confidence interval [CI]: 0.02, 0.33; p < 0.05) grades higher as compared with control group individuals from the same household. In the subsample of women, intervention group women attained 0.29 (95% CI: 0.04, 0.54; p < 0.05) more schooling grades as compared with control group women from the same household. Among unmarried women, those in the intervention group attained 0.32 (95% CI: 0.006, 0.63; p < 0.05) additional schooling grades as compared with the control group.

**Table 3:** Estimated association between the UIP and schooling attainment of Indians born in 1980–1995 (20–36 year old), analysis by subsamples.

	Estimated coefficient of intervention status				Sample size
	Household fixed-effects regression	Village or city ward fixed-effects regression	District fixed-effects regression	State fixed-effects regression	
All men and women of age 20–36 years	0.18* (0.08)	0.23* (0.05)	0.29** (0.05)	0.25** (0.07)	190,568
*Analysis by sex:*					
Men of age 20–36 years	−0.03 (0.12)	0.01 (0.07)	0.07 (0.07)	−0.03 (0.07)	73,511
Women of age 20–36 years	0.29* (0.13)	0.37** (0.07)	0.42** (0.07)	0.42** (0.1)	117,057
Unmarried women of age 20–36 years	0.32* (0.16)	0.33** (0.11)	0.38** (0.1)	0.45** (0.15)	46,847
*Analysis by location:*					
Members of rural households	0.16 (0.1)	0.19** (0.06)	0.28** (0.06)	0.27* (0.09)	136,663
Members of urban households	0.19 (0.12)	0.3** (0.08)	0.3** (0.08)	0.2* (0.09)	53,905
*Analysis by caste:*					
General caste	0.11 (0.16)	0.56** (0.11)	0.56** (0.11)	0.44** (0.14)	22,247
SC or ST	0.15 (0.13)	0.15 (0.08)	0.17* (0.07)	0.21 (0.12)	43,578
OBC	0.18 (0.13)	0.19* (0.09)	0.26** (0.08)	0.19* (0.08)	37,533
*Analysis by wealth quintile groups:*					
Members of households in the first (poorest) wealth quintile	0.22 (0.23)	0.16 (0.14)	0.23* (0.12)	0.31* (0.13)	20,192
Members of households in the fifth (richest) wealth quintile	0.21 (0.12)	0.32** (0.09)	0.26** (0.09)	0.18 (0.1)	26,233

Note: Data are from the National Family Health Survey of India 2015–2016. The sample consists of 20–36 year old men and women, all of whom had lived in the same location since birth. Only the estimated regression coefficient of intervention status (whether born when or after the UIP was implemented in the district) from each model is shown. The covariates of the models were age in years, squared age, sex (whether female), marital status (whether single), indicators of relationship to household head, location, caste groups, religion, and wealth quintiles, as applicable. Clustered standard errors are shown in parentheses. *P < 0.05, **P < 0.01.

### 3.3 Village or city ward fixed-effects regression results

In village or city ward fixed-effects analysis, intervention group individuals attained 0.23 (95% CI: 0.13, 0.32; p < 0.001) additional schooling grades as compared with the control group. Among women and unmarried women, those in the intervention group attained 0.37 (95% CI: 0.23, 0.5; p < 0.001) and 0.33 (95% CI: 0.12, 0.53; p < 0.01) additional schooling grades, respectively, as compared with the control group. Among members of households in rural areas and urban areas, and households from general caste, OBC, and those in wealth quintile 5, schooling attainment in the intervention group was 0.19 (95% CI: 0.08, 0.3; p < 0.001), 0.3 (95% CI: 0.13, 0.46; p < 0.001), 0.56 (95% CI: 0.34, 0.77; p < 0.001), 0.19 (95% CI: 0, 0.36; p < 0.05), and 0.32 (95% CI: 0.13, 0.5; p < 0.001) grades higher, respectively, as compared with the control group.

### 3.4 District fixed-effects regression results

In district fixed-effects analysis, the intervention group attained 0.29 (95% CI: 0.19, 0.38; p < 0.001) more schooling grades as compared with the control group. Among women and unmarried women, the estimated associations of the UIP were 0.42 (95% CI: 0.28, 0.55; p < 0.001) and 0.38 (95% CI: 0.18, 0.57; p < 0.001) additional schooling grades, respectively.

Among members of rural and urban households, those in the intervention group attained 0.28 (95% CI: 0.16, 0.38; p < 0.001) and 0.3 (95% CI: 0.14, 0.46; p < 0.001) additional schooling grades, respectively, as compared with the control group. Among members of general caste, SC or ST, and OBC households, intervention group adults attained 0.56 (95% CI: 0.34, 0.76; p < 0.001), 0.17 (95% CI: 0.02, 0.31; p < 0.05), and 0.26 (95% CI: 0.1, 0.42; p < 0.01) extra schooling grades, respectively, as compared with the control group. In households in the poorest and richest wealth quintiles, the intervention group attained and 0.26 (95% CI: 0.08, 0.43; p < 0.01) more schooling grades respectively as compared with the control group.

### 3.5 State fixed-effects regression results

Finally, in state fixed-effects analysis, individuals in the intervention group attained 0.25 (95% CI: 0.1, 0.39; p < 0.01) more schooling grades as compared with the control group. Among women and unmarried women, the intervention group attained 0.42 (95% CI: 0.21, 0.62; p < 0.001) and 0.45 (95% CI: 0.15, 0.74; p < 0.01) additional schooling grades, respectively, as compared with the control group.

Among members of rural and urban households, those in the intervention group attained 0.27 (95% CI: 0.09, 0.44; p < 0.01) and 0.2 (95% CI: 0.02, 0.36; p < 0.05) additional schooling grades, respectively, as compared with the control group. In households that belonged to general caste, OBC, and the poorest wealth quintile, intervention group adults attained 0.44 (95% CI: 0.17, 0.7; p < 0.01), 0.19 (95% CI: 0.02, 0.35; p < 0.05), and 0.31 (95% CI: 0.04, 0.57; p < 0.05) extra schooling grades, respectively, as compared with the control group.

### 3.6 Results from regression with expanded age range

Table 4 presents the estimates from the analysis of adult women of age 18–49 years and adult men of age 18–54 years, which are also summarized here. The estimated associations of the UIP with schooling attainment were positive and statistically significant across all subsamples and regression models, except for the subsample of men in state fixed-effects analysis where it was not significant.

**Table 4:** Estimated association between the UIP and schooling attainment of Indian women of age 18–49 years and men of age 18–54 years, analysis by subsamples.

	Estimated coefficient of intervention status				Sample size
	Household fixed-effects regression	Village or city ward fixed-effects regression	District fixed-effects regression	State fixed-effects regression	
All men and women	0.64** (0.06)	0.62** (0.04)	0.68** (0.05)	0.65** (0.13)	190,568
*Analysis by sex:*					
Men of age 18–54 years	0.40** (0.09)	0.24** (0.06)	0.27** (0.06)	0.19 (0.09)	73,511
Women of age 18–49 years	0.84** (0.1)	0.94** (0.06)	1.02** (0.08)	1.00** (0.17)	117,057
Unmarried women of age 18–49 years	0.97** (0.14)	1.14** (0.09)	1.20** (0.10)	1.16** (0.19)	77,603
*Analysis by location:*					
Members of rural households	0.57** (0.08)	0.59** (0.05)	0.69** (0.06)	0.68** (0.16)	136,663
Members of urban households	0.75** (0.1)	0.66** (0.07)	0.66** (0.08)	0.60** (0.09)	53,905
*Analysis by caste:*					
General caste	0.79** (0.13)	1.05** (0.09)	1.04** (0.10)	0.96** (0.18)	38,058
SC or ST	0.39** (0.10)	0.40** (0.06)	0.47** (0.07)	0.50** (0.15)	75,084
OBC	0.73** (0.11)	0.64** (0.07)	0.74** (0.08)	0.67** (0.15)	66,880
*Analysis by wealth quintile groups:*					
Members of households in the first (poorest) wealth quintile	−0.06 (0.2)	0.19 (0.12)	0.36** (0.13)	0.46 (0.28)	28,558
Members of households in the fifth (richest) wealth quintile	0.75** (0.11)	0.85** (0.08)	0.82** (0.08)	0.73** (0.14)	41,555

Note: Data are from the National Family Health Survey of India 2015–2016. The sample consists of 18–49 year old women and 18–54 year old men, all of whom had lived in the same location since birth. Only the estimated regression coefficient of intervention status (whether born when or after the UIP was implemented in the district) from each model is shown. The covariates of the models were age in years, squared age, sex (whether female), marital status (whether single), indicators of relationship to household head, location, caste groups, religion, and wealth quintiles, as applicable. Clustered standard errors are shown in parentheses. *P < 0.05, **P < 0.01.

The estimated associations of the UIP in the full sample of men and women ranged from 0.62 to 0.68 additional schooling grades. Among men, women, and unmarried women, it ranged from 0.24 to 0.4, 0.84 to 1.02, and 0.97 to 1.12 additional schooling grades, respectively. Among members of rural areas, urban areas, general caste, SC or ST, and OBC households, the estimated association ranged between 0.57 and 0.69, 0.6 and 0.75, 0.79 and 1.05, 0.39 and 0.5, and 0.64 and 0.74 more schooling grades, respectively. Finally, among members of households in the poorest wealth quintile, the estimated associations was 0.36, while it ranged between 0.73 and 0.85 in the richest wealth quintile.

## 4 Discussion

Vaccines could bring a wide range of benefits including improvements in health, schooling, economic productivity, equity, and aggregate macroeconomic indicators, along with reductions in healthcare costs and antimicrobial use. The benefits have been encapsulated by the broader economic impact of vaccination (BEIV) framework of the Initiative for Vaccine Research at the WHO (Jit et al., 2015). Table 5 provides an illustration of the framework.

**Table 5:** A framework for examining the broader benefits of vaccines.

Category	Definition	Outcome measures
A. Health-related benefits to vaccinated individuals		
A1. Health gains	Reduction in morbidity and mortality	Cases averted
		Deaths averted
		QALYs/DALYs saved
A2. Health care cost savings	Reduction in direct cost of health care borne by the public sector or private individuals	Costs saved by health care provider
		Health care costs saved by individuals
B. Productivity-related benefits		
B1. Productivity gains related to care	Reduction in lost days of work due to caring for a sick patient	Value of productivity
B2. Productivity gains related to health effects	Reduction in lost days of work due to sickness or death of a sick patient	Friction costs
		Potential lifetime earnings
B3. Productivity gains related to non-utility capabilities^a^	Increased lifetime productivity because of enhanced capabilities (such as improved cognition and educational attainment) not easily measured using utility-based preference measures	Educational outcomes
		Cognitive outcomes
		Potential lifetime earnings
C. Community or health systems externalities		
C1. Ecological effects	Health improvements in unvaccinated community members as a result of ecological effects such as herd immunity, eradication, and reduced antibiotic usage	Indirect vaccine protection
		Prevalence of antibiotic resistance
		Future cost of disease control averted
C2. Equity	More equal distribution of health outcomes	Distribution of health outcomes
C3. Financial and programmatic synergies and sustainability	Improved financial sustainability as a result of effects such as synergies with other health care programmes (e.g. delivery platforms), stimulation of private demand, and mechanisms to enhance group purchasing power (e.g. PAHO revolving fund)	Financial benefits
		Private demand estimates
C4. Household security	Improved financial security of households as a result of reduced risk of catastrophic expenditure	Actuarial value of security
D. Broader economic indicators		
D1. Changes to household behaviour	Economic improvements due to changes in household choices such as fertility and consumption/saving as a result of improved child health and survival	Productivity
		Female labour participation
		Household investment
		Child dependency ratio
D2. Public sector budget impact	Change to an individual's net transfers to the national budget over his/her lifetime	Return on investment
		Net present value of investment
D3. Short-term macroeconomic impact	Changes to national income or production as a result of short-term exogeneous shocks to the economy	Change in GDP (per capita)
		Change in sectoral output
D4. Long-term macroeconomic impact	Changes to national income or production as a result of long-term changes to drivers such as labour supply and foreign direct investment	Change in GDP (per capita)

We examined the long-term schooling benefits of childhood vaccination in India, using data from administrative sources and a large national household survey. We found that nonmigrant 20–36 year old adults who were born when or after the Universal Immunization Programme was implemented in their district of residence attained 0.18–0.29 more grades of schooling, as compared with those born before the UIP. The estimates—which represented intent to treat, i.e., exposure to the UIP—were similar across four different fixed-effects regression models. In subsample analysis, the estimates were statistically significant among women, unmarried women, members of households in rural and urban areas, general caste and OBC, and those in the top two wealth quintiles. Exposure to the UIP was associated with 0.6–0.7 extra grades of schooling when we expanded the age range to include all nonmigrant 18–49 year old women and 18–54 year old men. The estimated associations were positive and statistically significant in almost all additional subsamples of this expanded age range.

The estimated positive associations were likely also linked with height. A large international literature associates infectious diseases in early childhood with stunting (Almond and Currie, 2011; Checkley et al., 2008; Currie and Vogl, 2013a; Dewey and Begum, 2011). A previous study in India has linked the UIP with 0.3–0.5 higher height-for-age and weight-for-age z-scores of children under 5 years of age (Anekwe and Kumar, 2012). Other studies of the measles and Hib vaccines in India have found 0.1–0.3 higher height-for-age z-scores and 0.1–0.2 more schooling grades attained among vaccinated children of age 7–15 years, as compared with similar unvaccinated children (Nandi et al., 2019c, 2019b).

The stronger associations of the UIP with schooling among women and unmarried women, as compared with men, align with previous research on early childhood development in India. Historically, human capital investment by parents, such as the provision of nutrition and healthcare, has been lower among young Indian girls than among boys (Jayachandran and Kuziemko, 2011; Oster, 2009). Public programs may, therefore, have a relatively larger positive effect among girls. For example, supplementary nutrition in early life provided by a large national nutritional program has been linked with higher schooling attainment among adolescent and adult women in India, as compared with men (Nandi et al., 2019a, 2018, 2016). Rates of full vaccination among Indian children under the age of 2 years in 1992–1993, the earliest year for which such data were available, were similar between boys (37%) and girls (34%) (International Institute for Population Sciences, 1995). Therefore, the associations of the UIP among women, who otherwise received fewer early childhood resources, are likely to be stronger.

Our results contribute to a broader literature on the long-term effects of childhood infections, or their prevention or treatment, on nonhealth outcomes (Currie and Vogl, 2013a). Aside from the previously discussed vaccine studies in India and other countries, studies have linked prenatal exposure to influenza with lower schooling attainment, employment rates, and wages in Brazil, Taiwan, the UK, and the USA (Almond, 2006; Kelly, 2011; Lin and Liu, 2014; Nelson, 2010). Malaria eradication has been associated with increases in literacy rates, schooling attainment, and household consumption in Brazil, Colombia, Mexico, Paraguay, Sri Lanka, and the USA (Bleakley, 2010; Lucas, 2010). Availability of sulfa drugs *in utero* and lower pneumonia burden have been linked with higher adult schooling, employment rates, and wages in the USA (Bhalotra and Venkataramani, 2011), deworming treatment among Kenyan schoolchildren has been linked with later life gains in schooling, test scores, working hours, and IQ (Currie and Vogl, 2013b), and a malaria eradication campaign has been associated with increases in schooling attainment and male wage employment rates in Uganda (Barofsky et al., 2015).

The findings have important policy implications. Although gross enrolment ratio in primary education in India was 98% in 2015–2016, 4% each of those enrolled in primary and upper primary levels dropped out, and 17% dropped out at the secondary level (Department of School Education and Literacy, Statistics Division, 2018). Among socioeconomically disadvantaged ST children, annual drop-out rates at primary, upper primary, and secondary levels were 7%, 9%, and 25%, respectively (Department of School Education and Literacy, Statistics Division, 2018). Gross enrolment ratio in college or equivalent level (ages 18–23 years) in India remains at only 25%, and the average number of school grades completed by Indian adults was just 6.6 years in 2016 (Department of School Education and Literacy, Statistics Division, 2018; International Institute for Population Sciences and ICF, 2017). Our findings indicate that childhood immunization could improve adult schooling attainment in India by as much as 10%.

Gains in schooling attainment could also translate into higher earnings. Each extra year of schooling in India is estimated to increase men's wages by 4–6% and women's wages by 5–8% (Fulford, 2014). In 2011–2012—the most recent year for which such data are available—the average daily wage among Indians aged 15–59 years was INR 247 (US$4.5, assuming US$1 = INR 55) (International Labour Organization, 2018). Among men, the average was INR 151–470 (US$2.7–8.5) based on employment type, while among women, it was INR 104–365 (US$1.9–6.6) (International Labour Organization, 2018). The UIP is also likely to improve the economic status of women in India. For example, among unmarried women, the UIP is associated with an increment of 1.2 schooling years, which corresponds to as much as an INR 35 (US$0.6) increase in daily wages.

While the effect of returns to schooling on gross domestic product (GDP) has not been previously computed in the context of India, a global study indicates that an extra year of schooling is associated with a 0.37% increase in global GDP growth (Hanushek et al., 2008). Applying this estimate to India, and considering that the Indian GDP in 2018 was $2.72 trillion (current US$), exposure to UIP in early life could contribute $2–3 billion to GDP (equivalent to 0.2–0.3 extra schooling years) (World Bank, 2018).

India's UIP aims to cover 27 million newborn children and 30 million pregnant women every year (Ministry of Health and Family Welfare, 2017). At present, the program provides the following childhood vaccines: BCG; measles; DPT; OPV; inactivated polio vaccine; hepatitis B; Hib-containing pentavalent (DPT, hepatitis B, and Hib); and in endemic areas, Japanese encephalitis. The rotavirus, pneumococcal, and measles–rubella vaccines have also been introduced in some areas (Chatterjee et al., 2018; Ministry of Health and Family Welfare, 2017). However, more efforts are required to meet coverage targets. In 2016, only 62% of Indian children of age 12–23 months were fully vaccinated (measles, BCG, and three doses each of polio and DPT) (IIPS, 2016). Large subnational disparities also existed: Full vaccination rates ranged from 35% in Nagaland to 89% in Puducherry (International Institute for Population Sciences and ICF, 2017). In larger states such as Assam, Gujarat, and Uttar Pradesh, less than 52% of children under the age of 2 years were fully vaccinated (International Institute for Population Sciences and ICF, 2017).

Our findings reemphasize the need for universal coverage of routine childhood vaccines in India. In 2015, the Government of India introduced a large supplementary immunization program named Mission Indradhanush, followed by another campaign called Intensified Mission Indradhanush in 2017. Together, these programs aimed to vaccinate 90% of Indian children under the age of 2 years with routine vaccines by the end of 2018. Preliminary evidence suggests that both programs raised coverage rates substantially, although the 90% target may not have been met (Gurnani et al., 2018; Pramanik et al., 2016).

To maintain the momentum generated by these supplementary programs and to reach and continue providing near-universal vaccination coverage in the future, more resources must be allocated to the UIP. A recent study has estimated that the annual budgetary deficit of the program increased from US$9 to US$544 million (2013 US$) in 2013–2017 (Chatterjee et al., 2016). As new vaccines are introduced and funding from external sources such as Gavi, the Vaccine Alliance—which supports 3% of the UIP's budget—are phased out, the budgetary shortfall may increase (Chatterjee et al., 2016).

Our analysis has some limitations. The age of adults and the time of the UIP rollout in a district were both reported in years. Some adults who were born during the year prior to when the UIP was implemented in their district may have been exposed to the program in the first year of life. Due to lack of data on months of birth and UIP implementation, we considered them to be in the control group. Moreover, although the UIP target group was children under the age of 1 year, older children—some of whom were in our control group— may also have been vaccinated. Therefore, the estimated positive associations of the UIP with schooling attainment may be conservative.

Although we accounted for many socioeconomic, demographic, and community-level indicators in our models, unobserved factors may be correlated with our posited determinants of educational status and with immunization. For example, parental decision to vaccinate a child may depend upon perceived health status or growth of the child, which were unobserved in our data. If healthier children were vaccinated at higher rates, the estimated positive associations between the UIP and schooling attainment may be inflated. Alternatively, if parents decided to provide vaccines to weaker children at higher rates, our results could be conservative.

Vaccines provide herd protection to unvaccinated people, especially those living in close proximity to vaccinated individuals, such as within the same household or village. The estimated positive associations between the UIP and schooling attainment may be conservative particularly in the household and village fixed-effect models, as the control group may have received unobserved secondary immunity. The characteristics of nonmigrants and migrants were statistically different, as shown in Table 2 and discussed earlier. Although the differences were small in magnitude, our findings may not be generalizable to migrants.

Despite such limitations, our results show that beyond immediate reduction in disease morbidity and mortality, routine childhood vaccines could also generate substantial long-term schooling and economic gains for India. The benefits are higher for women, narrowing the male-female gap in schooling and wages. The findings reemphasize the need for universal coverage of routine childhood vaccines among Indian children.

## CRediT authorship contribution statement

**Arindam Nandi:** Methodology, Data curation, Formal analysis, Writing - original draft, Writing - review & editing. **Santosh Kumar:** Methodology, Data curation, Writing - review & editing. **Anita Shet:** Writing - review & editing. **David E. Bloom:** Writing - review & editing. **Ramanan Laxminarayan:** Methodology, Writing - review & editing.

## Declaration of competing interest

DB has previously received research support or personal fees from GlaxoSmithKline plc, Merck, Pfizer, and Sanofi-Pasteur related generally to value-of-vaccination research, but not for this study. All other authors declare no conflict of interest.

## References

[bib1] Almond D. (2006). Is the 1918 influenza pandemic over? Long‐term effects of in utero influenza exposure in the post‐1940 U.S. Population. J. Polit. Econ..

[bib2] Almond D., Currie J. (2011). Killing me softly: the fetal origins hypothesis. J. Econ. Perspect..

[bib3] Anekwe T.D., Kumar S. (2012). The effect of a vaccination program on child anthropometry: evidence from India's Universal Immunization Program. J. Public Health.

[bib4] Anekwe T.D., Newell M.-L., Tanser F., Pillay D., Bärnighausen T. (2015). The causal effect of childhood measles vaccination on educational attainment: a mother fixed-effects study in rural South Africa. Vaccine.

[bib5] Barofsky J., Anekwe T.D., Chase C. (2015). Malaria eradication and economic outcomes in sub-Saharan Africa: evidence from Uganda. J. Health Econ..

[bib6] Bhalotra S.R., Venkataramani A. (2011). The Captain of the Men of Death and His Shadow: Long-Run Impacts of Early Life Pneumonia Exposure (SSRN Scholarly Paper No. ID 1951332).

[bib7] Bleakley H. (2010). Malaria eradication in the Americas: a retrospective analysis of childhood exposure. Am. Econ. J. Appl. Econ..

[bib8] Bloom D.E., Canning D., Shenoy E.S. (2012). The effect of vaccination on children's physical and cognitive development in the Philippines. Appl. Econ..

[bib9] Canning D., Razzaque A., Driessen J., Walker D.G., Streatfield P.K., Yunus M. (2011). The effect of maternal tetanus immunization on children's schooling attainment in Matlab, Bangladesh: follow-up of a randomized trial. Soc. Sci. Med..

[bib10] Chadda R.K., Deb K.S. (2013). Indian family systems, collectivistic society and psychotherapy. Indian J. Psychiatr..

[bib11] Chatterjee S., Das P., Nigam A., Nandi A., Brenzel L., Ray A., Haldar P., Aggarwal M.K., Laxminarayan R. (2018). Variation in cost and performance of routine immunisation service delivery in India. BMJ Glob. Health.

[bib12] Chatterjee S., Pant M., Haldar P., Aggarwal M.K., Laxminarayan R. (2016). Current costs & projected financial needs of India's Universal Immunization Programme. Indian J. Med. Res..

[bib13] Checkley W., Buckley G., Gilman R.H., Assis A.M., Guerrant R.L., Morris S.S., Mølbak K., Valentiner-Branth P., Lanata C.F., Black R.E. (2008). Multi-country analysis of the effects of diarrhoea on childhood stunting. Int. J. Epidemiol..

[bib14] Currie J., Vogl T. (2013). Early-life health and adult circumstance in developing countries. Ann. Rev. Econ..

[bib15] Currie J., Vogl T. (2013). Early-life health and adult circumstance in developing countries. Ann. Rev. Econ..

[bib16] Department of School Education and Literacy, Statistics Division (2018). Department of School Education and Literacy, Statistics Division Educational Statistics at a Glance.

[bib17] Dewey K.G., Begum K. (2011). Long-term consequences of stunting in early life. Matern. Child Nutr..

[bib18] Driessen J., Razzaque A., Walker D., Canning D. (2015). The effect of childhood measles vaccination on school enrolment in Matlab, Bangladesh. Appl. Econ..

[bib19] Filmer D., Pritchett L.H. (2001). Estimating wealth effects without expenditure data-or-tears: an application to educational enrollments in States of India. Demography.

[bib20] Fulford S. (2014).

[bib21] Government of India (2019). Districts of India [WWW Document]. Government of India Web Directory. http://www.goidirectory.gov.in/district.php.

[bib22] Gurnani V., Haldar P., Aggarwal M.K., Das M.K., Chauhan A., Murray J., Arora N.K., Jhalani M., Sudan P. (2018). Improving vaccination coverage in India: lessons from Intensified Mission Indradhanush, a cross-sectoral systems strengthening strategy. BMJ.

[bib23] Hanushek E.A., Jamison D.T., Jamison E.A., Woessmann L. (2008). Education and economic growth: it's not just going to school but learning that matters. Educ. Next.

[bib24] IIPS (2016). National Family Health Survey 4 (NFHS-4): India Fact Sheet.

[bib25] International Institute for Population Sciences (1995). National Family Health Survey 1992-1993.

[bib26] International Institute for Population Sciences, ICF (2017). National Family Health Survey (NFHS-4) 2015-2016: India.

[bib27] International Labour Organization (2018). India Wage Report: Wage Policies for Decent Work and Inclusive Growth.

[bib28] Jayachandran S., Kuziemko I. (2011). Why do mothers breastfeed girls less than boys? Evidence and implications for child health in India. Q. J. Econ..

[bib29] Jit M., Hutubessy R., Png M.E., Sundaram N., Audimulam J., Salim S., Yoong J. (2015). The broader economic impact of vaccination: reviewing and appraising the strength of evidence. BMC Med..

[bib30] Kelly E. (2011). The scourge of Asian flu: in utero exposure to pandemic influenza and the development of a cohort of British children. J. Hum. Resour..

[bib31] Kumar S. (2009). Childhood Immunization, Mortality and Human Capital Accumulation: Micro-evidence from India (MPRA Paper).

[bib32] Lahariya C. (2014). A brief history of vaccines & vaccination in India. Indian J. Med. Res..

[bib33] Lin M.-J., Liu E.M. (2014). Does in utero exposure to Illness matter? The 1918 influenza epidemic in Taiwan as a natural experiment. J. Health Econ..

[bib34] Lucas A.M. (2010). Malaria eradication and educational attainment: evidence from Paraguay and Sri Lanka. Am. Econ. J. Appl. Econ..

[bib35] Ministry of Health & Family Welfare (2017). Immunization Handbook for Medical Officers.

[bib36] Ministry of Health and Family Welfare (2017). Intensified Mission Indradhanush Operational Guidelines.

[bib37] Nandi A., Ashok A., Kinra S., Behrman J.R., Laxminarayan R. (2016). Early childhood nutrition is positively associated with adolescent educational outcomes: evidence from the Andhra Pradesh child and parents study (APCAPS). J. Nutr..

[bib38] Nandi A., Behrman J., Laxminarayan R. (2019). The Impact of a National Early Childhood Development Program on Future Schooling Attainment: Evidence from ICDS in India.

[bib39] Nandi A., Behrman J.R., Kinra S., Laxminarayan R. (2018). Early-life nutrition is associated positively with schooling and labor market outcomes and negatively with marriage rates at age 20–25 Years: evidence from the Andhra Pradesh children and parents study (APCAPS) in India. J. Nutr..

[bib40] Nandi A., Deolalikar A.B., Bloom D.E., Laxminarayan R. (2019). Haemophilus influenzae type b vaccination and anthropometric, cognitive, and schooling outcomes among Indian children. Ann. N. Y. Acad. Sci..

[bib41] Nandi A., Shet A. (2020). Why Vaccines Matter: Understanding the Broader Health, Economic, and Child Development Benefits of Routine Vaccination.

[bib42] Nandi A., Shet A., Behrman J.R., Black M.M., Bloom D.E., Laxminarayan R. (2019). Anthropometric, cognitive, and schooling benefits of measles vaccination: longitudinal cohort analysis in Ethiopia, India, and Vietnam. Vaccine.

[bib43] Nelson R.E. (2010). Testing the fetal origins hypothesis in a developing country: evidence from the 1918 influenza pandemic. Health Econ..

[bib44] Office of the Registrar General and Census Commissioner (2011). Census of India 2011 [WWW Document]. Government of India. http://censusindia.gov.in/2011-Common/CensusData2011.html.

[bib45] Office of the Registrar General and Census Commissioner, India, n.d. Census of India (2001). General Population Tables (Tables A-1 to A-3).

[bib46] Oster E. (2009). Proximate sources of population sex imbalance in India. Demography.

[bib47] Ozawa S., Mirelman A., Stack M.L., Walker D.G., Levine O.S. (2012). Cost-effectiveness and economic benefits of vaccines in low- and middle-income countries: a systematic review. Vaccine.

[bib48] Pollitt E., Gorman K.S., Engle P.L., Martorell R., Rivera J. (1993). Early supplementary feeding and cognition: effects over two decades. Monogr. Soc. Res. Child Dev..

[bib49] Pramanik S., Agrahari K., Srivastava A., Varanasi V., Setia M., Laxminarayan R. (2016). Integrated Child Health and Immunization Survey, Rounds 1 & 2 Report.

[bib50] Upadhyay A.K., Srivastava S. (2017). Association between Haemophilus influenza type B (Hib) vaccination and child anthropometric outcomes in Andhra Pradesh (India): evidence from the young lives study. J. Public Health.

[bib51] Vashishtha V.M., Kumar P. (2013). 50 years of immunization in India: progress and future. Indian Pediatr..

[bib52] World Bank (2018). GDP, PPP (Current International $) - India [WWW Document]. World Bank. https://data.worldbank.org/indicator/NY.GDP.MKTP.PP.CD?locations=IN.

[bib53] World Health Organization (2018). World Health Organization: 10 Facts on Immunization [WWW Document]. https://www.who.int/features/factfiles/immunization/en/.

[bib54] You D., Hug L., Ejdemyr S., Idele P., Hogan D., Mathers C., Gerland P., New J.R., Alkema L. (2015). Global, regional, and national levels and trends in under-5 mortality between 1990 and 2015, with scenario-based projections to 2030: a systematic analysis by the UN Inter-agency Group for Child Mortality Estimation. Lancet.

